# Anatomical basis for the choice of laparoscopic surgery for low rectal cancer through the pelvic imaging data—a cohort study

**DOI:** 10.1186/s12957-018-1498-z

**Published:** 2018-10-05

**Authors:** Zhou Yang, Guo Chunhua, Yuan Huayan, Yang Jianguo, Cheng Yong

**Affiliations:** Department of Gastrointestinal Surgery, First Affiliated Hospital of Chongqing Medical University, Chongqing, 400010 China

**Keywords:** Low rectal cancer, Laparoscopic surgery, Sphincter preservation, Pelvic measurement

## Abstract

**Background:**

Low rectal cancer surgery without anus conservation needs permanent ileostomy or colostomy which seriously affects the quality of life of patients. Therefore, low rectal cancer surgery not only pays attention to the safety of surgical treatment but also to the anus conservation.

**Methods:**

Sixty-seven patients suffering from low rectal cancer had undergone laparoscopic surgery which was analyzed through retrospective study. They were divided into the anus-conserving and non-anus-conserving groups. Thirty-five set of pelvic data was obtained from the preoperative CT and MRI images. After that, the discriminant function was obtained to predict the surgery methods for patients with low rectal carcinoma.

**Results:**

Anal-conserving group discriminant function (F1) = − 33.698 + 6.045 × anal margin distance (cm) + 1.105 × T4; non-anus-conserving group discriminant function (F2) = − 14.125 + 3.138 × anal margin distance (cm) + 0.804 × T4. If F1 is greater than F2, then the case can be treated as the anus reservation while if F2 is greater than F1 the case cannot be treated anus reservation. The accuracy of the discriminant function was evaluated which was found to be 97%.

**Conclusion:**

The discriminant function of pelvic data provides anatomical basis for the choice of surgical methods for low rectal cancer.

## Background

Colorectal cancers are due to old age and lifestyle factors. There are several ways to treat rectal cancer depending on its type and stage which includes surgery, radiation therapy, and ablation or embolization therapy. Surgery plays a curative role for early rectal cancer. In recent years, there are many surgical procedures for the treatment of rectal cancer which includes Hartmann (proctosigmoidectomy), Dixon, Miles, TaTME, and ISR. But the total mesorectal excision (TME) is still the gold standard for rectal cancer surgery [1]. Apart from this, laparoscopic surgery as new techniques has been evolved for the treatment of rectal cancer to reduce postoperative complication [2]. It is widely used because of its rapid recovery of intestinal function, little surgical trauma, less pain, and hemorrhage as well as the short time of hospitalization. Moreover, prospective randomized studies showed that the laparoscopic surgery as compared with the traditional laparotomy does not affect the surgical outcomes [3, 4].

In our present study, considering no cell invasion at lower border of tumor, the length of incisional margin, 5-year survival rate and local recurrence of rectal carcinoma had not showed any significant correlation which is currently the pathological basis of anus-conserving surgery. Preoperative chemoradiotherapy helps to enhance the chance of anus-conserving surgery by 10%–20% [5, 6]. It aims to reduce the size or extent of the cancer before using radical treatment intervention thus making procedures easier and more likely to succeed. Low rectal cancer surgery without anus conservation needs permanent ileostomy or colostomy which seriously affects the quality of life of patients. Therefore, low rectal cancer surgery not only pays attention to the safety of surgical treatment but also to the anus conservation [7].

Although, laparoscopic surgery has drawn more attention among the patients and doctors, the indication of laparoscopic surgery with anus conservation have been the focus of controversy about which the majority of surgeons believe that they are relevant with the tumor location, size, stage, patient gender, obesity, and other [8, 9]. The handling of this new technique requires good experience. Thus, more experienced surgeons can easily do laparoscopic low rectal cancer surgery with anus conservation, [10] but it is technically difficult for young surgeons. In this study, we found that the deep and narrow pelvis is positively associated with operation time and bleeding volume [11, 12]. Moreover, the sacrum angle and pelvic measurements were associated with making preoperative decision in TME surgery [13]**.** Is there any relationship between the choice of laparoscopic surgery for rectal cancer and pelvic measurements? What kind of pelvis can allow laparoscopic anus-conserving surgery? The answer to this question has been explained through our study. In this study, 67 rectal cancer patients undergoing laparoscopic surgery were examined by CT three-dimensional reconstruction and MRI images, and the relationship between pelvic data and the choice of laparoscopic rectal cancer surgery was studied.

## Methods

### Patients

This study had been reported in line with the STROCSS criteria [14] which included 67 cases of colonoscopy-confirmed rectal cancer patients from the anal margin less than 7 cm from August 2015 to April 2017 at department of gastrointestinal surgery of our university by the same senior surgeon. The work was retrospective and single-center. Patients with stage T4, preoperative manifestation of intestinal obstruction or perforation, tumor distance from the anal greater than 7 cm, patients after neoadjuvant chemoradiotherapy, and those who underwent laparotomy were excluded. According to the operation information of the patients, the patients were divided into anus-conserving and non-anus-conserving groups. The laparoscopic resection was performed in the anus-conserving group, and the MILES was performed in the non-anus-conserving group. Table 1 showed the clinical features and tumor characteristics of these patients. This study was approved by the medical ethics committee of Chongqing Medical University (CMU-2016501a). Informed consent was obtained from the patients for publication of this paper and any accompanying images.

**Table 1:** Clinical and tumor characteristics of the patients

	Operation methods		*P*
	Anal reservation(48)	Non-anal reservation(19)	
Age	62.38	57.84	0.230
BMI	23.36	22.65	0.359
Gender			
Male (43)	33	10	0.215
Female (24)	15	9	
Anal distance(cm)	5.286	2.587	0.000
Complication			
With (24)	15	6	0.979
Without(46)	33	13	
Tumor staging			
T1 (3)	3	0	0.128
T2 (25)	15	10	
T3 (39)	30	9	
Transverse diameter of tumor (cm)	3.417	3.425	0.651
Tumor location			
Front wall (5)	2	3	0.386
Left wall (32)	26	6	
Right wall (30)	20	10	

*Anal distance*, the distance from the lower edge of the tumor to the anus was measured along the central axis of the pelvic MRI. *Transverse diameter of tumor*, the maximum width of coronal plane was measured by MRI

### Surgical methods

Rectal cancer patients with preoperative evaluation without surgical contraindication and with the consent of the patient and family members, laparoscopic resection was carried out by using following two methods of surgery.

Specific steps of laparoscopic anterior resection of rectal cancer are as follows: inserted five Trocar with the navel for observation hole, left middle abdomen and lower abdomen for assistant holes, and right middle abdominal and lower abdomen for operation and ultrasonic knife hole. Made the lithotomy position with trendelenburg and high left low right position, and then established 10–15 mmHg CO_2_ pneumoperitoneum to push the small intestine to right upper quadrant. And then the inferior mesenteric artery trunk was separated along sigmoid mesocolon root and the inferior mesenteric artery and vein were ligated. Along the Toldts gap, the bowel was separated to sigmoid colon and descending colon junction where, according to the TME principle to isolate intestine to down edge of tumor and intraluminal stapler was used for mutilation of intestinal tube. Stopped pneumoperitoneum, taken out the separated intestinal tube from abdominal cavity by making a 4 cm oblique incision at the left lower abdomen and cut the intestinal tube at the safe distance from tumor. Pouch suturing intestinal tube and embedding anastomat were made and pneumoperitoneum was re-established. Intestinal anastomosis was performed by DST technique. According to the situation of operation, ileostomy or/and transverse colostomy were made.

MILES: surgical procedures are same with above-mentioned rectal cancer resection. But after mutilation of intestinal tube and stopped pneumoperitoneum, a 2.5–3 cm circular incision was made in the left at the first/third junction of an imaginary line between left anterior superior spine and umbilicus. Taken out, sigmoid colon cut the intestinal tube at proximal 15 cm from tumor, and sigmoid colostomy was made. In the perineal operation, a shuttle incision was made at sciatic tuberosities, midline of the perineum, and the apex of the coccyx to protect the external genitalia and the subcutaneous tissue and each layer were separated, and the anus and rectum were removed, and anastomosis was made with abdominal operation.

Free dissection of pelvic visceral fascia, internal iliac vascular sheath and paravascular lymph nodes, lymphatic tissue in the bladder cavity, and routine pathological examination of excised specimens were carried out after surgery.

### Pelvic measurement

**Fig. 1:**
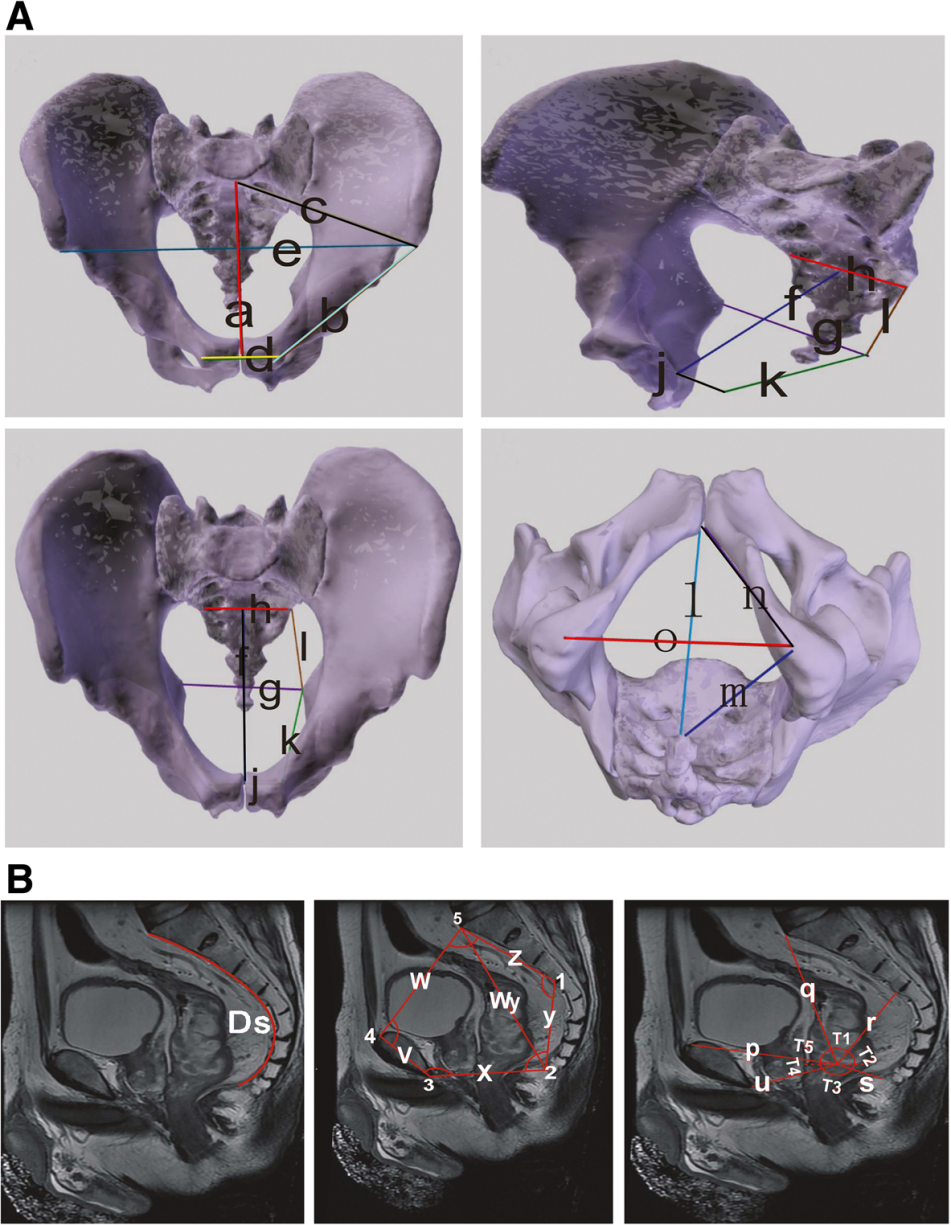
**a** Definition of pelvic parameters assayed by CT. Pelvic inlet plane: (a) anteroposterior diameter of entrance; (b) diameter between anterior superior iliac spine and pubic bone; (c) diameter between anterior superior iliac spine and sacrum; (d) superior border diameter of pubis tubercle; (e) anterior superior iliac spine diameter. Middle pelvic plane: (f) anteroposterior diameter of middle pelvis; (g) bispinous diameter; (h) S3 width; (i) diameter of S3 and ischial spine; (j) midline diameter of pubis; (k) diameter between pubis and spinal ischiadica. Pelvic outlet plane**:** (l) diameter between pubic bone and coccyx; (m) diameter between ischial tuberosity and coccyx; (n) diameter between pubic and ishial tuberosity; (o) diameter of ischial tuberosity. **b** Definition of pelvic parameters assayed by MRI. (v) Suprapubic diameter; (z) sacral S3; (Wy) sacrococcygeal diameter; (Ds) caudal arc length; (y) S3 tail; (q) sacral T; (r) T-S3; (s) T tail; (p) T suprapubic; (u) T subpubic; (1) the angle between the vertex of sacral promontory and the apex of the coccyx; (2) the angle between the lower edge of the third sacral bone and the lower margin of the pubic bone; (3) the angle between the apex of the coccyx and the upper margin of pubis; (4) the angle between the vertex of sacral promontory and the lower margin of the pubic bone; (5) the angle between the vertex of sacral promontory and the lower edge of the third sacral bone; (T1) the angle between the vertex of sacral promontory and the lower edge of the third sacral bone; (T2) the angle between the lower edge of the third sacral tubercle and the apex of the coccyx; (T3) the angle between the apex of the coccyx and the lower border of the pubic bone; (T4) the angle between the lower border and upper border of the pubic bone; (T5) the angle between the upper border of the pubic bone and the vertex of sacral promontory

CT and MRI are relatively accurate methods for pelvic measurement [15, 16]. In preoperative evaluation, the abdominal CT and pelvic MRI examination of all 67 patients included in this study were carried out (CT model: 64 slice spiral CT, Light Speed VCT; MRI machine: MAGNETOM Avanto) and ADW 4.4 workstation was used for 3D processing of CT scan image, and 3D image construction. The pelvic MRI scan images were processed with PACS software. ADW 4.4 workstation was used to measure the parameters of the pelvis, including the pelvic entrance plane, the middle pelvic plane, and the pelvic outlet plane. The sagittal plane of the pelvis was measured with PACS software in MRI images. The measurements were completed by two radiologists without knowing the patient’s clinical information. Specific measurement parameters are shown in Fig. 1.

The operation methods as the dependent variable, the single factor analysis were used to find the correlation of specific measurement parameters and operation methods. The statistically significant variables were then analyzed by discriminant analysis and used to establish prediction equations.

### Statistical analysis

Excel 2003 was used to sort out the data, and SPSS 21 software was used for statistical analysis. The test data for normal distribution is checked by Shapiro-Wilk test. In univariate analysis, for the normal distribution of data, the relationship between variables and operation methods was analyzed by independent samples *t* test; for non-normal distribution data, Mann-Whitney *U* (rank) was used to examine the relationship between the study variables and operation methods. After single factor analysis showed the statistical significances, Fisher (step) analysis method was used to further study of the influencing factors of surgery, establish the discriminant function, and verify the accuracy of discriminant function (*P* < 0.05 indicated significant).

## Results

In this study, 19 patients underwent Miles surgery to remove the anus, and 48 patients underwent laparoscopic anterior resection to keep the anus. The average age of the anus-conserving group was 62.38, and the non-anus-conserving was 57.84. And the mean BMI of the anus-conserving group was less than that of the non-anus-conserving group, and the anus-conserving group in the T3 phase was significantly more than that in the non-anus-conserving group. The BMI, gender, tumor staging, tumor transverse diameter, and tumor location had no effects on the surgical procedure (*P* > 0.05). The average distance of the anus-conserving group from the anal margin was 5.286 cm while of the non-anus-conserving group was 2.587 cm, which had significant difference (*P* = 0.000). See Table 1.

The operation methods as the dependent variable, the single factor analysis was used to find that anal margin (cm), suprapubic inter diameter, S3 width, sacral arc length, sacral S3, sacral T, T-S3, T tail, Subpubic T, T1, T2, T3, T4 a total of 13 independent variables were statistical significance (*P* < 0.05). See Table 2. The 13 statistically significant variables were then analyzed by discriminant analysis, and the results showed that the anal margin (cm), T4 was statistically significant for surgery methods (Wilks, lambda ≥ 0.38, *P* < 0.001). Fisher discriminant function: anus-conserving group discriminant function (F1) = − 33.698 + 6.045 × anal margin distance (cm) + 1.105 × T4; non-anus-conserving group discriminant function (F2) = − 14.125 + 3.138 × anal margin distance (cm) + 0.804 × T4. If F1 is greater than F2, the patients in anatomy should be treated as the anus reservation, if F2 is greater than F1, the patients in anatomy should be treated as non-anus reservation.

**Table 2:** The association between measurement parameters and surgical methods

Parameters	Mean ± SD (min, max) or median (four quantile interval)	*F* or *Z*	*P*
Transverse diameter of tumor (cm)	3.363 (2.903, 3.825)	− 0.452	0.651
Anal margin distance (cm)	4.868 (3.20, 5.69)	− 6.065	0.000
a	10.97 ± 1.15 (8.20, 14.2)	− 0.108	0.914
b	13.4 ± 0.83 (11.67, 15.85)	1.392	0.169
c	12.48 (12.17, 13.06)	− 0.814	0.416
d	7.43 (6.48, 7.80)	− 2.024	0.043
e	22.83 ± 1.63 (18.89, 26.51)	1.979	0.052
f	11.71 (11.24, 12.49)	− 0.549	0.583
g	10.00 (9.12, 10.92)	− 1.273	0.203
h	8.25 (7.92, 8.60)	− 2.003	0.045
i	7.08 ± 0.87 (5.43, 9.29)	−0.805	0.424
j	5.32 ± 0.54 (4.39, 6.54)	1.625	0.109
k	8.92 ± 0.51 (7.82, 10.37)	0.497	0.621
l	8.53 ± 0.81 (6.73, 10.92)	− 0.947	0.347
m	5.93 ± 0.97 (4.05, 8.16)	− 0.595	0.554
n	9.13 ± 0.99 (6.43, 11.18)	0.366	0.716
o	11.04 ± 1.52 (7.57, 14.27)	0.389	0.698
Ds	15.04 ± 1.12 (12.98, 17.60)	3.013	0.004
v	5.00 (4.76, 5.34)	− 0.529	0.597
Wy	11.83 ± 1.13 (9.16, 13.9)	1.914	0.06
z	7.76 ± 0.53 (6.36, 8.87)	2.738	0.008
y	6.27 ± 0.79 (4.21, 8.14)	1.951	0.055
1	114.14 ± 8.09 (93.37, 129.20)	− 0.243	0.809
2	111.86 (32.55, 67.81)	− 0.108	0.914
3	124.15 (141.48, 189.50)	− 0.16	0.873
4	96.76 ± 7.25 (81.86, 113.87)	1.102	0.274
5	91.14 ± 7.99 (72.27, 113.12)	− 0.92	0.361
q	11.30 ± 1.51 (7.80, 14.78)	− 3.505	0.001
r	8.25 (7.31, 9.37)	− 6.525	0.000
s	4.05 (3.25, 4.92)	− 4.674	0.000
p	8.58 ± 1.01 (6.32, 11.18)	1.663	0.101
u	4.53 (3.94, 5.33)	− 3.297	0.001
T1	7.78 (38.1, 48.14)	− 5.008	0.000
T2	43.43 (32.55, 67.81)	− 5.133	0.000
T3	169.11 (141.48, 189.50)	− 5.606	0.000
T4	28.70 ± 6.55 (13.31, 40.27)	4.937	0.000
T5	63.59 (59.78, 71.72)	2.676	0.009

Table 3 showed that in the anus-conserving group, 44 of the 48 cases were correctly judged, and 18 of the 19 cases in the non-anus-conserving group were correctly judged, that is, 92.5% of the patients were correctly operated on. The accuracies of discriminant function for anus-conserving and non-conserving groups were 91.67% and 94.74%, respectively.

**Table 3:** The accuracies of the discriminant function

Actual operations	Prediction		Total
	Anus reservation	Non-anus reservation	
Anus reservation	44 (91.67%)	4	48
Non-anus reservation	1	18 (94.74%)	19

## Discussion

The third most common type of cancer is rectal cancer worldwide with increasing incidence rate. Surgical resection is the preferred curative approach. Laparoscopic surgery is being used widely as compared to open surgery. With the development of surgical techniques, surgeons realized that the narrow space of the pelvis significantly restricted the operation of rectal cancer surgery, and even affected the surgical outcomes [17–19]. The nature of the tumor, the extent of the disease, and anatomical description of disease should be established prior to surgery. Pelvimeter is no longer just obstetric assessment tool, but widely used in the evaluation of rectal cancer before surgery. A lot of researches have proved that there is a link between the pelvic measurement parameters and operation time, intraoperative bleeding volume, difficulty of operation, ring margin positive rate, surgical specimen integrity, even postoperative anastomotic fistula of rectal cancer surgery [11–13, 20]. In this study, two methods of laparoscopic surgery was used, one was laparoscopic anterior resection in which the part of the rectum containing the tumor was removed and the colon was then attached to the remaining part of the rectum so that bowels can be empty in the usual way, second was MILES which involved removal of the anus and the tissues surrounding it, including the sphincter muscle and a permanent colostomy was done since the anus was removed. DST technique is considered the standard method of the rectum anastomosis [21], and we think the linear cutter into the safe distance under rectal cancer may be affected by the pelvic structure, which may affect the surgery at anatomy level.

In the 35 set of measured parameters, 10 set of them are obviously related with the location of tumor, but at the same time the structure of the whole pelvis, which can obviously reflect the relationship between the pelvic position and rectal tumor. By univariate analysis, there was statistical significance between 13 independent variables and the operation methods, and discriminant function showed that anal margin distance and T4 have significant influence on the methods of operation. Patients with larger T4 may be more likely to keep their anus, and the author thinks that this may be related to the extending angle of the linear cutter. However, in discriminant function, the distance between the tumor and the anal margin is significantly larger than the coefficient of T4, that is to say, the distance between the tumor and the anal margin is still the decisive factor for anus reservation or not.

However, this study of 67 patients in BMI (*P* = 0.359), gender (*P* = 0.215), tumor diameter (*P* = 0.651), tumor location (*P* = 0.386), and tumor stage (*P* = 0.128) had no statistical significance in the anus reservation or not, which are not consistent with other research [8, 9]. In this study, we focus on the relationship between pelvic measurement parameters and operation methods, the accuracy of function F1 and F2 for prediction of operation methods of were 91.67% and 94.74% respectively. Therefore, it was concluded that the function F1 and F2 may simply predict the surgical methods from the pelvic anatomy level. However, the accuracies of discriminant function for anus reservation or not need to be validated on larger samples.

## Conclusion

Through this study, we found that T4 plays an important role in the lower rectal cancer surgical options. The predictive accuracy of functional F1 and F2 was more than 90%. Combining with the general condition of patients and tumor, assessment of pelvic anatomy level will help to decide anus reservation or not for the low rectal cancer surgery.
